# Case of Irritation of the Urethra and Neck of the Bladder Arising from Stricture of the Rectum

**Published:** 1825-07

**Authors:** W. White


### Art. IV.—*Case of Irritation of the Urethra and Neck of the Bladder arising from Stricture of the Rectum.* By W. White, Esq.

During the month of September last, a captain of a merchant-vessel, about forty-five years of age, applied to me, complaining that he had been afflicted upwards of two years with violent spasms in the course of the urethra, accompanied with considerable difficulty in making water. For this complaint he had not been able to obtain the smallest relief; though he had consulted several medical gentlemen, amongst whom were some of the first surgeons of the metropolis. From a suspicion of there being a stone in the bladder, he was sounded by one of those gentlemen, who decidedly gave his opinion that this was not the case. To judge from what he prescribed for the patient, which was chiefly opium, he evidently considered the complaint as one of irritability and spasm; but from which treatment he did not derive any benefit. Another surgeon in London was afterwards consulted, who passed a bougie into the bladder, and pronounced the disease to be a stricture of the urethra. However, as that gentleman did not advise the use of a bougie, but merely recommended the occasional employment of a warm bath, and other means that were calculated to allay local irritation, it is very probable that he also considered the stricture entirely spasmodic. Notwithstanding, the patient experienced no abatement of his sufferings from the remedial means employed: and in this situation he was under the necessity of undertaking a voyage to sea, when he was necessarily deprived, for several months, of all medical assistance. During this period, his complaint became more aggravated. Soon after his return, business led him to visit Bristol, where he embraced the opportunity of consulting another surgeon, who, suspecting* him to labour under a stricture of the rectum, examined the intestine, when he was convinced of the existence of that disease. As the patient passed through Bath, on his way to London, the surgeon advised him to consult me. On my introducing a bougie, I found the rectum extremely irritable, and

---

* The obstinate state of the bowels led to a suspicion of the existence of the disease; and this the other surgeons had not attended to.

much contracted. There were two strictures; the first about four inches from the extremity, the other between seven and eight inches; which, with difficulty, admitted the passing of a seventh-size bougie. He afterwards proceeded on his journey to London; but, in the course of a short time, returned to Bath, and placed himself under my care.

His general health and appetite were very good, but his bowels had been in a constipated state for several years: he seldom had an evacuation oftener than once in three or four days; and not even then without taking some active aperient medicine. He had not noticed having passed a figured motion for a long time, and, when he did so, they appeared to be very small and flat. After every alvine evacuation, the spasms of the urethra came on, though by no means so severe as at the time of, and after, making water; an inclination to which he often felt (almost every ten minutes) during the day, but only discharged a few drops at a time. Sometimes, indeed, the spasms were so severe, that he was under the necessity of sitting up nearly the whole of the night in bed, with the *pot de chambre* between his legs, occasionally passing a few drops of urine, which were sometimes followed by a little blood; so that his situation was truly distressing.

On the 27th of September, a seventh-size return bougie was introduced, attended with much irritation. The patient was directed to take a small quantity of castor-oil every night, to keep the bowels open.

October 2d.—The bougie has been daily employed, and the spasms are rather less severe after making water. The bowels have been open the last two days, without the necessity of taking any castor-oil; a circumstance that had not once happened since he had been afflicted with the complaint.

Oct. 4th.—Has had rather more of the spasms to-day; the bougie was therefore omitted.

Oct. 6th.—The spasms being less severe, the bougie was again introduced. It is rather singular that the patient experiences more freedom from spasm during the time the bougie remains in the rectum, than any other part of the day.

Oct. 7th.—He had no return of spasm yesterday for several hours after using the bougie; but in the evening experienced rather a sharp attack, which lasted only a short time.

Oct. 8th.—The former part of yesterday, he passed his urine unattended by spasm; he had likewise less inclination to micturition. Took a little castor-oil last night, which operated freely. Bougie introduced.

Oct. 9th.—Had rather more of the spasms yesterday, which were most probably owing to the bowels being disturbed, by the castor-oil operating rather too briskly.

Oct. 11th.—Has been altogether free from the spasm, but felt a sharp pricking pain in the urethra this morning, after making water, which he describes as being different from spasm.

Oct. 20th.—The bougie has been daily employed, and there has been a gradual diminution of the spasms. Instead of being disturbed at least a dozen times in the course of the night to pass water, he is no longer under the necessity of rising oftener than six or seven times, and then micturition is only attended with a pricking sensation: he is afterwards enabled to lie down, and go to sleep immediately.

Oct. 23d.—He still experiences less inclination to make water, as well as less suffering afterwards. Bougie introduced.

Oct. 25th.—The bougie was omitted yesterday, too much irritation having been excited after its last introduction. To-day it was passed, but the patient was requested not to keep it in quite so long.

Oct. 26th.—Considerably less irritation from the introduction of the last bougie. Had a natural motion this morning. Bougie introduced.

Oct. 27th.—Feels better to-day than he has done yet. Took a little castor-oil last night, which procured two motions this morning, without the least pain or spasm.

Oct. 28th.—Had no return of spasm till four o'clock this morning, when he experienced a sharp attack for a short time after making water.

Oct. 30th.—He retained the bougie four hours yesterday: it was, however, omitted to-day, as too much irritation had been excited.

November 1st.—There being less irritation to-day, the bougie was introduced.

Nov. 5th.—He passed water yesterday without any return of the spasms, till about five o'clock in the evening, when he had another sharp attack. Has had two natural motions this morning, so that the bowels are now able to perform their natural function with very little assistance, having had no occasion to take any castor-oil for five days. Bougie introduced.

Nov. 7th.—To-day he complains of pain about the left iliac region, evidently following the course of the colon: he was, therefore, advised to take a dose of castor-oil, as there was probably some fœcal accumulation; for, although the motions were regular, they had been rather scanty. Bougie omitted.

Nov. 8th.—The castor-oil operated freely. The pain which he complained of yesterday about the left iliac region, is entirely removed. The spasms are diminishing both in frequency and severity. The size of the bougie has been gradually increased, and the passage now admits of the third size being easily introduced.

Nov. 11th.—He retained the bougie yesterday three hours, without any increased uneasiness. He only passed water twice during this time, and that very freely, unattended with spasm.

Nov. 13th.—The second-size bougie was introduced, which passed very freely. He was able to retain it in the rectum four hours and a half. Felt very little of the spasms during the day afterwards.

Nov. 14th.—As he had rather more of the spasms, (probably owing to the increased size of the bougie, and his retaining it too long, though he did not feel any particular uneasiness at the time,) the bougie was not introduced. Had a natural motion yesterday, and also this morning, without pain.

Nov. 15th.—As the increased irritation had subsided, the bougie was again passed. The patient was desired not to retain it quite so long as the last time.

Nov. 16th.—Retained the bougie two hours yesterday; passing his urine, during this time, very freely, and without spasm. Had an evacuation in the evening, attended with a trifling discharge of bloody mucus. (This he had been accustomed to, but not since the use of the bougie.) As his bowels were rather confined, he was requested to take a dose of castor-oil. The bougie to-day passed more readily.

Nov. 17th.—He passed no water for more than two hours after introducing the bougie yesterday, which he retained three hours and a half.

Nov. 18th.—The bougie was retained only two hours, as he felt rather more irritation, though the spasms were not worse. He slept much longer last night without being disturbed to make water, than he has done yet. Had a motion this morning of a natural figure, and of equal size with the bougie.

Nov. 20th.—The patient is now more clearly convinced of the connexion between the obstructions of the rectum and the spasmodic state of the urethra, and of the former being the cause of the latter; for he finds that, when the bougie presses against the stricture,* the spasms of the urethra are instantly induced, but, so soon as the point of the instrument passes completely through the stricture, they subside. This effect is the more obvious since the bougie has been enlarged.

The same plan was persevered in till the 24th of December: the patient then being able to introduce the largest bougie very well himself, (the passing of which had nearly ceased to produce the spasms of the urethra,) he left Bath in a very comfortable state, having obtained considerable relief, which he had hitherto sought in vain. He was, however, requested to continue the use of the bougie some time longer, with a view of more effectually establishing the benefit he had derived from its application. For it may be observed, that in many cases a recurrence of the complaint has been experienced, where the person had been inattentive to the directions that were given, and leaving off the bougie too soon.

Although it is evident that the nature of the disease was understood in this case, from the remedial means which were employed, yet the cause whence the spasms originated was entirely overlooked, until the patient went to Bristol. It had been previously ascertained there was no stone in the bladder, by a surgeon whose judgment could not be called in question. This case points out the necessity of a careful investigation,

* Particularly the first.

that symptoms of a disease may not be mistaken for the disease itself.

P.S.—A few days since I received a letter from the gentleman, dated on-board a vessel at sea, expressive of his gratitude for the benefit he had experienced; at the same time acknowledging that he had not been so attentive to himself as he ought, from having been so busily engaged in fitting out his ship.

*Bath; March 25th,* 1825.

